# Take-Over Intention during Conditionally Automated Driving in China: Current Situation and Influencing Factors

**DOI:** 10.3390/ijerph182111076

**Published:** 2021-10-21

**Authors:** Zhongxiang Feng, Jingyu Li, Xiaoqin Xu, Amy Guo, Congjun Huang, Xu Jiang

**Affiliations:** 1 School of Transportation, Southeast University, Nanjing 210096, China; fzx@hfut.edu.cn; 2 School of Civil and Hydraulic Engineering, Hefei University of Technology, Hefei 230009, China; 3 School of Automobile and Traffic Engineering, Hefei University of Technology, Hefei 230009, China; xuxiaoqin@smedi.com; 4 Jiangsu Key Laboratory of Urban ITS, Southeast University, Nanjing 210096, China; amywhg@hotmail.com; 5 Jiangsu Province Collaborative Innovation Center of Modern Urban Traffic Technologies, Nanjing 210096, China; 6 Hefei Urban Planning and Design Institute, Hefei 230009, China; hcj13965051002@outlook.com (C.H.); jiangxu_524@live.com (X.J.)

**Keywords:** drivers, automated driving, take-over intention, technology acceptance, self-efficacy, risk perception

## Abstract

Drivers’ take-over intention is important for the design of the automated driving systems and affects the safety of automated driving. This study explored the influence factors on drivers’ take-over intention during conditionally automated driving, examined the correlations among factors through path analysis, and established a take-over intention model. A questionnaire survey was conducted in Hefei, China, and a sample of 277 drivers was obtained. Our study shows that the average take-over intention of those aged under 20 is lower than that of the older age groups. In the positive emotions (PE) scenarios, the take-over intention of aged 31–40 is significantly higher than that of the other age groups. Education and occupation have a significant influence on the take-over intention. The perceived ease of use (PEofU) and perceived usefulness (PU) of automated driving are significantly negatively correlated with drivers’ take-over intention in the road conditions (RC) and climate conditions (CC) scenarios. In addition, through path model analysis, our study shows that trust in the safety of autonomous vehicles (AVs) plays an important role in drivers’ take-over intention. Technology acceptance, risk perception and self-efficacy has indirectly correlated with take-over intention through trust in the safety of AVs. In general, drivers with lower technology acceptance, lower self-efficacy and higher risk perception are less likely to trust automated driving technology and have shown stronger intention to take-over the control of the vehicles.

## 1. Introduction

Automated driving technology is not only a technological innovation but also a breakthrough solution to some key problems in the traffic environment. For example, it has potential to prevent fatal collisions caused by human drivers’ error, provide personal transportation services for elderly and disabled individuals without the need of a driver, conserves energy and reduces emissions through potential reduction in car ownership and mileages [1,2,3]. The Society of Automotive Engineers divides automated driving technology into six levels: no automated driving (level 0), driver assistance (level 1), partial automated driving (level 2), conditional automated driving (level 3), high automated driving (level 4) and full automated driving (level 5) [4]. At present, driving assistance systems such as adaptive cruise control and parking assistance are widely available. Some automobile manufacturers (such as CHANA) claim that high-tech AVs will soon enter the market, and the public awareness of the fast development of AV technology is on the rise. However, as with any emerging technology, AVs can also be considered an innovative system with uncertainty, complexity, dynamics and not fully reliable and safe [5,6]. In SAE level 3, drivers or users need to take-over the control of the vehicle from the automated driving system in the event of an emergency, for example, when automated driving systems failure suddenly or situations that are beyond the ability of automated driving systems.

Safe take-over by human driver has been a key issue for AVs. A considerable amount of research on this subject has been conducted worldwide. Many have focused on the choices of take-over requests and take-over timing [7,8,9]. Some studies suggest voice alert [10,11] while others recommend visual and tactile schemes [12,13]. Bazilinskyy studied 2669 participants from 95 countries and found that gender and speech speed can influence the take-over time [14]. A series of driving simulator studies investigated the effectiveness of tri-modal (visual, auditory, and vibrotactile) displays on driver behavior and concluded that they increased the perceived urgency and perceived alerting effectiveness as compared to unimodal displays [15,16,17].

Investigating take-over behavior performance is not limited to take-over requests only. Blommer used a strategy of repeatedly demanding drivers to take over the control of the vehicle and found that intermittent manual driving reduced participants’ take-over response time [18]. KöRber explored age differences in taking over the control to avoid collisions using simulation experiments and concluded that age had an impact on driving behavior following the taking over but not the take-over time [19]. Gold demonstrated that traffic density increased the complexity of take-over tasks and affected drivers’ performance [20]. Boelhouwer examined how influential the information manual of an automated driving system was on driver’s take-over decisions [21]. The study suggested that the information manual provided no support on drivers’ performance and drivers needed to be guided based on individual preferences and specific situations to make correct take-over decisions.

In summary, take-over process has been studied intensively, with many focusing on investigating the methods to improve the take-over performance. However, whether a driver is willing to take-over or not and what are the influencing factors of such a decision-making process have not been explored sufficiently. In AV operation, it is possible that, if the system is not properly designed, the driver and the system would seek for or relinquish the control of the vehicle at the same time. That would cause severe crashes. California DMV 2018 Autonomous Vehicle Disengagement Reports pointed out that, out of the 70,165 manual interventions by Uber, 64,363 were “preventive take-over” made by drivers due to “excessive caution” (accounting for as much as 92.12%) [22]. We believe that drivers’ willingness to take-over should not be ignored. A better understanding of the factors influencing drivers’ take-over intention and take-over decision can assist with the design of safe, efficient and more user-friendly automated driving systems and improve the public’s trust in AVs.

In the present study, an AV take-over intention model was proposed and verified through exploring factors influencing driver’s decision on whether to reclaim the control of the SAE level 3 AVs under different scenarios. The technology acceptance model (TAM) was extended to establish a new path model. Driver’s self-efficacy and risk perception ability were added as possible influencing factors. Data were collected via a questionnaire survey. The influence degree of demographic factors on take-over intention, the path of influence of variables such as trust in safety, technology acceptance, risk perception and self-efficacy on AV take-over intention were explored. This study is the first attempt to extend the TAM to research on AV take-over intention. Trust in safety is a key mediating variable in the research hypothesis.

## 2. Presentation and Hypotheses of the Model

### 2.1. Extended Technology Acceptance Model

TAM defines user’s technology acceptance as their decision to use the technology [23]. The essence of take-over intention is that, in the automated driving mode, the driver decides whether to let the automated driving technology to be in control under the event of an emergency. Zhang’s acceptance model verifies the important influence of trust on drivers’ acceptance of the automated driving technology by adding initial trust (as a mediating variable) and risk perception factors (including security risk perception and privacy risk perception) to the original TAM [24]. The research results prove that initial trust and safety risk perception affect the use of automated driving technology.

TAM includes two main factors: perceived usefulness (PU), i.e., benefits the system provides, and perceived ease of use (PEofU), i.e., how easy to use the system. TAM is widely applied to explore drivers’ attitudes toward AVs [25,26]. Kaur argues that trust and PU are the two factors that directly influence the acceptance of AVs [27], while Zhang’s claims that PU has a positive impact on the trust in AV while PEofU does not [24].

**Figure 1:**
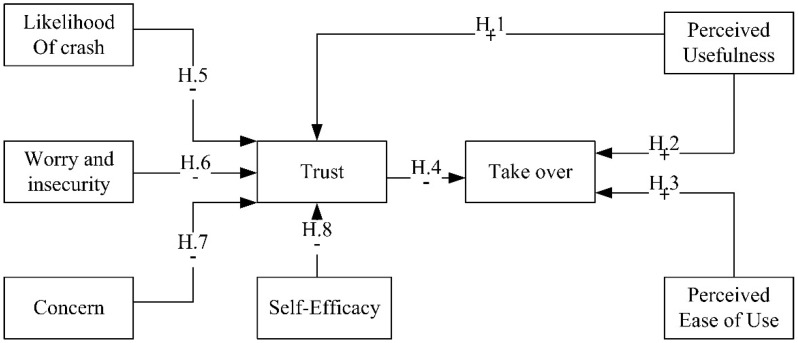
Diagram of the hypothesized model (Hypothesis 1–8). Notes: +: positive impact −: negative impact.

Based on this situation, the following hypotheses were established (as shown in Figure 1).

**Hypothesis** **1** **(H1).** *PU has a positive impact on trust in AVs*.

**Hypothesis** **2** **(H2).** *PU has a positive impact on drivers’ take-over intention*.

**Hypothesis** **3** **(H3).** *PEofU has a positive impact on drivers’ take-over intention*.

### 2.2. Technology Acceptance and the Take-Over Decision

Trust in AVs plays an important role in public acceptance and determining whether drivers adopt the technology and how much they can rely on it [28]. To date, the majority of AVs are unable to cope with all conditions yet; thus, drivers must be ready to take-over when needed. Being able to detect potential hazards is an important prerequisite for drivers to be well-prepared for take-over operation [29]. The take-over process includes three steps: discovering the potential hazard, comprehending the complexity of the situation, and finally, deciding whether to take-over. At this point, there could be two possible scenarios: the driver is not taking over because they believe that the automated driving technology can handle the situation safely; or the driver is taking over the control because they do not have sufficient trust in the AV. Some studies have shown that users could become over-dependent on the automated driving technology and totally detach themselves from monitoring the road and the driving task [30]. As a result, they lost their vigilance and ultimately led to collisions. Therefore, it is reasonable to believe that drivers’ trust in the safety of automated driving technology can influence their take-over decision under certain driving scenarios.

Thus, the following hypothesis was established:

**Hypothesis** **4** **(H4).** *Trust in the safety of AVs has a negative impact on drivers’ take-over intention*.

### 2.3. Risk Perception and Self-Efficacy

In the traffic environment, studies have proven that driver’s risk perception affects their choice of driving speed and the way in which people choose to travel [31]. Research on automated driving technology shows that people still have some concerns about the safety-related risks of using AVs [32]. The risk perception of AVs refers to the driver’s cognition and judgment of possible accident risk, which has been proven to be a key factor of trust in technology [33]. This paper argues that drivers’ risk perception could be one of the key factors affecting trust in automated driving technology. It was evaluated through a subjective evaluation scale, and its sub-factors include the likelihood of a crash and concern (cognition-based risk perception) and worry and insecurity (emotion-based risk perception) (see 3.2.5). Therefore, the following hypotheses were established:

**Hypothesis** **5** **(H5).** *The likelihood of a crash has a negative impact on trust in the safety of AVs*.

**Hypothesis** **6** **(H6).** *Worry and insecurity have a negative impact on trust in the safety of AVs*.

**Hypothesis** **7** **(H7).** *Concern over accidents has a negative impact on trust in the safety of AVs*.

In addition, the driver’s self-assessment of driving ability could become an influencing factor that affects the trust in automated driving technology. Self-efficacy is defined as an individual’s subjective evaluation of their ability to organize and execute the action process needed to achieve goals [34]. The evaluation results directly affect people’s behavioral motivation. The driver’s self-efficacy is the subjective evaluation of his or her driving skills, and the evaluation of his or her driving skills is the embodiment of the driver’s trust in themselves. Self-driving technology cannot handle every emergency yet; therefore, drivers judge the reliability of automated driving technology based on their own driving technology cognition. However, the specific situation needs to be further explored. It is assumed that the driver’s self-efficacy affects their trust in the technology and, the driver’s take-over intention. Therefore, the following hypothesis was established:

**Hypothesis** **8** **(H8).** *Self-efficacy has a negative impact on trust in the safety of AVs*.

## 3. Methods

### 3.1. Participants

The questionnaire survey was conducted from October to November 2018. Drivers that were waiting for services in the car maintenance stores (Volkswagen, Mercedes-Benz, Volvo, Chana and other mainstream automobile brands in the Chinese market) in Hefei, Anhui province, China, were selected to participate in the survey. All questionnaires were distributed in the form of printout. Prior to their participation, all were informed about the purpose of the survey, their freedom to withdrawn at any time, and the data collected from them would be stored anonymously. Upon obtaining their consent, a 28 s video was played to briefly introduce the concept of AV. The first part of the video showed an AV driving with Chinese dubbing. The video screen was split into two parts vertically with the driver’s main perspective on the left and the driver’s view of the three mirrors on the right. The second part contained two scenarios of an AV avoiding dangerous situations (1) at night, the AV comes to a full stop to avoid left-turning vehicles in the opposite lane; (2) at an interstate ramp in daylight, the AV slows down whilst turning right and then stops to avoid tailgating the front vehicle that has come to a sudden stop.

Among the total of 285 completed questionnaires, 277 were valid with 226 male (81.6%) and 51 female (18.4%), which is in line with the national gender distribution of drivers in China [35]. The age of the participants ranged from 18 to 57 years old (M = 28.3, SD = 8.6) and they were divided into four age groups: ≤20, 21–30, 31–40, ≥41 with the understanding that drivers aged 20–40 are at a peak of their driving ability [36]. Their driving experience ranged from 1 to 35 years (M = 5, SD = 5.8). The demographic data of the samples are shown in Table 1.

### 3.2. Materials

The questionnaire used in this study was divided into five parts: demographic information, the Automated driving Take-Over Intention Scale (ADTIS), the Technology Acceptance Scale (TAS), the Adelaide Driving Self-Efficacy Scale (ADSES) and the Risk Perception Scale (RPS).

#### 3.2.1. Demographic Questionnaire

The participants were required to provide the following personal information: gender, age, driving experience, educational level, number of traffic accidents in the past three years, experience with a driving assistance system and a 5-point Likert scale was employed to understand their level of trust in the safety of automated driving: from “1”, do not trust the system at all, to “5′”, trust completely.

#### 3.2.2. Automated Driving Take-Over Intention Scale

There are 29 questions in the ADTIS and a 5-point Likert scale was applied. A lower score represents a higher level of intention to take-over the control of the vehicle from the automated driving system. The questions on the scale are concise and easy for drivers to understand and respond. The design of the questionnaire is based on those take-over scenarios employed in existing experimental research under two considerations [37,38]. The first consideration was the location of the incident that could have an impact on the driver’s intention to take-over, where a construction site and a pedestrians crossing had been the most chosen sites in previous studies. The second consideration was the driver’s own state that could affect their intention to take-over, such as fatigue. The process of scale analysis and verification is shown in Section 3.3.1.

#### 3.2.3. Technology Acceptance Scale

This study used the automated driving TAS compiled by Buckley [28]. The TAS contains two subscales with a total of 12 items to which participants respond on a 7-point Likert scale (1 “completely disagree” to 7 “completely agree”). In this study, the scale was revised (see 2.3.4 for the revision process), and the Chinese version of the TAS was established by exploratory factor analysis (EFA) and confirmatory factor analysis (CFA); 9 questions were asked, and 2 subscales were applied: PU (alpha = 0.86) and PEofU (alpha = 0.85).

#### 3.2.4. Adelaide Driving Self-Efficacy Scale

This study used the ADSES, which was developed by George (2007) and proved to be effective in evaluating driving self-efficacy (alpha = 0.946) [39]. The ADSES has a total of 12 items and uses a 7-point Likert scale (1 “completely disagree” to 7 “completely agree”).

#### 3.2.5. Risk Perception Scale

Rundmo (2004) argues that the RPS for drivers includes three dimensions: likelihood of a crash, worry and insecurity, and concern [40]. The 10-item RPS used in this study was developed by Ma (2009) [41]. The RPS mainly evaluates worry and insecurity (alpha = 0.875), likelihood of a crash (alpha = 0.857), and concern (alpha = 0.871), and uses a 5-point Likert scale (1 “very unlikely/not concerned” to 5 “very likely/concerned”).

### 3.3. Statistical Analysis

In this study, a total of 285 drivers participated in the survey. Four questionnaires were eliminated because they were incomplete or incorrectly completed, and an additional 4 questionnaires were eliminated because the response to the question “How many years have you been driving” was “0”. Hence data collected from a total of 277 questionnaires were used for the final analysis.

#### 3.3.1. Exploratory Factor Analysis

EFA was conducted on the 29 items of the ADTIS, and principal component analysis and orthogonal rotation were adopted because the Kaiser–Meyer–Olkin (KMO) statistic is 0.887, greater than 0.70, and the result of Bartlett’s test of sphericity was significant (*p* < 0.001) (add a reference). Five common factors with eigenvalues greater than 1 were extracted, and the cumulative variance contribution rate was 72.961%. To ensure at least 3 questions for each factor and a factor loading higher than 0.40, 7 questions (Q6, Q7, Q8, Q9, Q11, Q17 and Q22) were deleted after three repeated comparisons.

Principal component analysis and orthogonal rotation were carried out again for the remaining 22 questions. The results showed that KMO = 0.861, and the result of Bartlett’s test of sphericity was significant (*p* < 0.001). Four common factors with eigenvalues greater than 1 were extracted, and the cumulative variance contribution rate was 69.781%. At the same time, the factor loading was greater than 0.55, indicating that the scale had good construct validity.

The factor loadings of each item are shown in Table 2. Factor 1 contains 5 items with contents such as “When it is raining heavily, I’m willing to take-over the control of the AV” and “When snow is falling, I’m willing to take-over the control of the AV”. All these items reflect the drivers’ willingness to take-over under certain climate conditions; thus, this subfactor is named climate conditions (CC). Factor 2 contains 9 items, such as “When a section of road has construction ahead, I’m willing to take-over the AV”. All these items reflect the drivers’ willingness to take-over under certain road conditions; thus, this subfactor is named road conditions (RC). Factor 3 contains 5 items with contents such as “When I’m upset, I’m willing to take-over the control of the AV”. All these items reflect the drivers’ willingness to take-over when they were in a negative state; thus, this subfactor is named negative states (NS). Factor 4 contains three items with contents such as “When I am in a good mood, I’m willing to take-over the AV”. All these items reflect the drivers’ willingness to take-over when they were experiencing positive emotions; thus, this subfactor is named positive emotions (PE).

#### 3.3.2. Reliability and Validity

The self-reported ADTIS scores from the sample ranges from 0 to 21. The reliability and validity of this part were analyzed, and all the Cronbach’s alpha values were greater than 0.85, indicating a strong reliability (see Table 3) and a good internal consistency of the questionnaire.

The validity of the questionnaire refers to the degree to which the measuring tool or instrument can accurately measure what is to be measured. The Pearson correlation coefficient was used to test the construct validity between the subscales and between the subscales and total scale. As shown in Table 4, there is a significant correlation between CC (M = 4.08, SD = 0.71), RC (M = 3.80, SD = 0.76), NS (M = 2.99, SD = 1.14) and PE (M = 2.68, SD = 0.97). They are also correlated with the total scale to a higher degree, which indicates that each subscale has better discriminant validity and that each subscale has better construct validity with the total scale.

#### 3.3.3. Confirmatory Factor Analysis

EFA focuses on testing the relationship between the assumed observed variables and the assumed potential variables. CFA tests the fit of the hypothesized structure. A four-factor original structure was built based on the EFA results: chi-square test, *p* < 0.001, Tucker–Lewis index (TLI) = 0.901, comparative fit index (CFI) = 0.913, and root mean square error of approximation (RMSEA) = 0.090. From the overall results, the model and data still have a good fit (as shown in Table 5).

#### 3.3.4. Scale Validity

Since the TAS used in this study comes from English-speaking countries, it was necessary to translate it into Chinese and verify its reliability and validity. Principal component analysis and orthogonal rotation were used to extract common factors with eigenvalues greater than 1 from the 12 items of the scale. As shown in Table 6, ultimately, two factors were rotated to obtain a total of 9 questions, and the cumulative variance contribution rate was 66.817%. The KMO statistic was 0.859 > 0.5, and the result of Bartlett’s test of sphericity was significant (*p* < 0.001). As shown in Table 6, these two factors are PU (M = 4.71, SD = 2.68; alpha = 0.86) and PEofU (M = 4.39, SD = 2.27; alpha = 0.85), and there is a significant correlation between them (r = 0.604 **, *p* < 0.001). Therefore, the 9-item TAS used in this study has good reliability and validity.

Since the ADSES used in this study comes from English-speaking countries and was translated into Chinese, a test of its reliability and validity of the scale was needed. Principal component analysis and orthogonal rotation were used to extract common factors with eigenvalues greater than 1 from the 12 items of the scale. Ultimately, self-efficacy (M = 6.961, SD = 5.32; alpha = 0.946) was obtained by rotation, and there were 12 questions in total. The variance contribution rate was 63.1%, the KMO statistic was 0.942 > 0.5, and the result of Bartlett’s test of sphericity was significant (*p* < 0.001). This suggests good reliability and validity of the 12-item self-efficacy scale in this study (as shown in Table 7).

The RPS was employed to evaluate drivers’ risk perception. As shown in Table 8, there is a significant correlation between the three subscales and the internal consistency coefficient (Cronbach’s alpha) values of the three subscales are all greater than 0.8, indicating a strong reliability. Therefore, the scale developed by Ma (2009) has good reliability and validity in this study [41].

## 4. Results

### 4.1. Technology Acceptance, Self-Efficacy, Risk Perception and AV Take-Over Intention

This study explored the correlations between the driver’s take-over intention and technology acceptance, self-efficacy, risk perception, drivers’ trust in AVs (see Table 9). The results show that PU and PEofU are correlated with drivers’ take-over intention in a significantly negative way. That is, those who are more likely to think that automated driving technology is useful and easy to use are less intended to take-over the control of the vehicle. The results also suggest that the likelihood of a crash increases the drivers’ take-over intention. However, their take-over intention does not seem to be significantly correlated with self-efficacy and risk perception. Among demographic factors, gender is significantly correlated with take-over intention only in the PE scores, age is significantly correlated with take-over intention in the NS scores, educational level is significantly correlated with take-over intention except in the CC scores, and driving experience is significantly correlated with take-over intention in the CC and NS scores.

As shown in Table 10, drivers’ trust in the AV system is correlated with the driver’s PU, PEofU and self-efficacy in a significantly positive way and with the likelihood of a crash in a significantly negative way, but is not correlated with other risk perception factors. In addition, there is no significant correlation between their trust in the safety of AVs and their take-over intention in the NS scores. This indicates that, when the likelihood of a crash increases, trust is weakened and, as a result, the drivers’ take-over intention becomes stronger. Meanwhile, when the driver is in a negative state, their intention to take-over and their trust in the system appear to be unrelated.

### 4.2. Demographic Variables and Driver Behaviors

One-way ANOVA was conducted on the results of the ADTIS and demographic variables including the drivers’ age and educational level. As shown in Table 11, there is an overall difference between the four age groups in NS and PE subscales. Multiple least significant difference (LSD) comparisons revealed no significant differences between the 21–30 group and the over-41 group; however, the drivers in the under-20 group had significantly higher self-reported NS scores than those in the other groups, and were less willing to take-over. The PE scores of the 31–40 group were significantly higher than those of the other groups. When compared with other groups, the 31–40 group had a stronger take-over intention.

In addition, the educational level is significantly correlated with RC, NS and PE. Further multiple LSD comparisons revealed no significant differences between those with a bachelor’s degree and those with a master’s degree or above. However, the self-reported scores of take-over intention from those without any degree were significantly higher than that from those with degrees. This suggests that drivers with higher education are less intended to take-over (as shown in Table 12).

### 4.3. Degree of Influence of Take-Over Intention, Technology Acceptance, Risk Perception and Self-Efficacy

**Figure 2:**
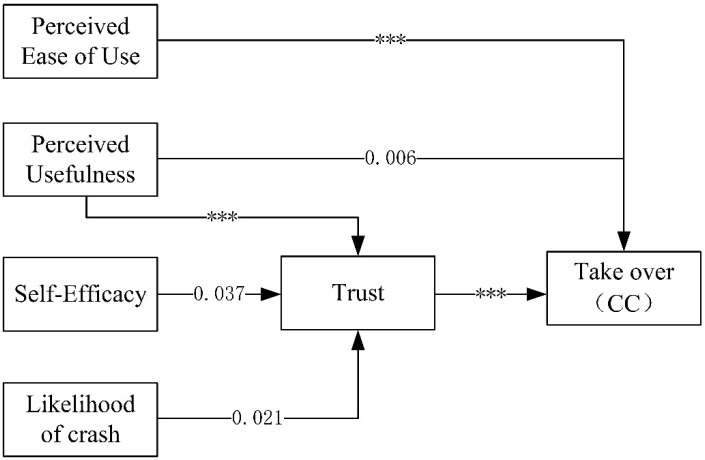
Value of CC model Notes: *** *p* < 0.001.

**Figure 3:**
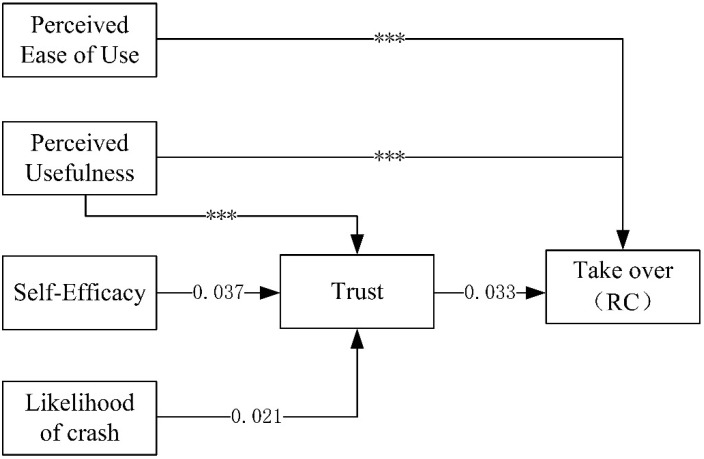
Value of RC model Notes: *** *p* < 0.001.

Neither worry and insecurity nor concern was significantly associated with trust (see Table 10). Thus, hypotheses 6 and 7 are eliminated from the model. To verify the hypotheses, a path model was established for the four subfactors of the ADTIS, and the fit of the model was evaluated. The results indicate that the path was significant only in the CC and RC scores (Figure 2 and Figure 3). The following criteria were applied to examine the fit of significant models: the chi-square test, CFI, TLI and RMSEA. The results show that the CC and RC models have a good fit (CC: chi-square = 7.783, DF = 3, *p* = 0.051, CFI = 0.981, TLI = 0.906, RMSEA = 0.076; RC: chi-square = 4.140, DF = 3, *p* = 0.247, CFI = 0.996, TLI = 0.979, RMSEA = 0.037).

The standardized path analysis results are shown in Table 13. Regarding the results of the CC model shown in Figure 2, the CC model assumes significant paths, and the statistically significant path results show that PEofU, PU and trust are directly negatively correlated with take-over intention. The path from PU to trust to take-over intention and the path from self-efficacy to trust to take-over intention are significantly correlated. Self-efficacy is only indirectly positively correlated with take-over intention. The path from likelihood of a crash to trust to take-over intention is statistically significantly correlated, and likelihood of a crash is indirectly negatively correlated with take-over intention. Regarding the results of the RC model, combined with Figure 3, overall, the results are similar to those of the CC model and prove the reliability of the CC and RC models. In the CC model, trust has a stronger influence on take-over intention. Therefore, hypothesis 4 and 5 are supported by the CC and RC models. However, the results do not support hypotheses 1, 2, 3 and 8, i.e., drivers with higher self-efficacy scores are more likely to trust AVs and are less likely to take-over.

## 5. Discussion

This study explored the influence of demographic variables (age, gender, occupation and educational level) and key factors (technology acceptance, self-efficacy, and risk perception) on drivers’ take-over intention in four scenarios. Meanwhile, Automated driving Take-over Intention Scale (ADTIS) was developed, the correlations among factors through path analysis were explored, and a take-over intention model was established. The results show that drivers have an active take-over intention under (in order from strongest to weakest) climate conditions (CC), road conditions (RC), negative states (NS) and positive emotions (PE) and their take-over intention vary in different take-over scenarios. There is a significant correlation between technology acceptance/trust and autonomous vehicle (AV) take-over intention. Self-efficacy and likelihood of a crash are correlated indirectly with the take-over intention through the influence of trust as a mediating variable. Demographic factors such as age, educational level and occupation all play a significant role in drivers’ take-over intention.

Studies by other scholars have shown that existing information manual of an automated driving system cannot provide drivers with sufficient support to decide whether to take-over the control of the vehicle [18]. There is a significant negative correlation between the subfactors (perceived usefulness (PU), perceived ease of use (PEofU)) of the technology acceptance model (TAM) and take-over intention in risk scenarios; risk perception and self-efficacy are not directly and significantly correlated with AV take-over intention. Therefore, the model of drivers’ take-over intention was established to explore the influencing factors of drivers’ take-over intention. The results suggest that there is a significant negative correlation between drivers’ trust in the safety of automated driving and their take-over intention, i.e., the more they trust the automated driving system, the lower the likelihood of taking over the control of the vehicle.

Our study also explored the relationship between demographic variables and AV take-over intention. One-way ANOVA found that there was no significant difference in take-over intention between those in their 20s and those aged over 40. The self-reported NS take-over intention scores of drivers aged 20 and under were significantly lower than those aged over 20. Drivers aged 31-40 had significantly higher PE scores than those in other groups, their average driving years was high and trusted their driving skills more than the functions of the automated driving system. In addition, significant correlations were found between educational level and the RC, NS and PE scores, but there was no significant difference in take-over intention across the four scenarios between the group with a bachelor’s degree and the group with a master’s degree or above. The self-reported scores of drivers without a degree were significantly higher than those with a degree, which suggests that they have a stronger take-over intention. It could be that drivers with higher educational level are more knowledgeable about automated driving technology, hence are more willing to trust such a technology.

A path model was established to explore the influence of various factors on AV take-over intention in depth, and led to the development of the take-over intention model. The results show that the paths of PU, PEofU, self-efficacy and likelihood of a crash to AV take-over intention are statistically significant (under RC and CC). In the model, trust is significantly directly correlated with take-over intention (the higher the trust score is, the greater the likelihood of drivers choosing automated driving instead of taking over). PU and PEofU are significantly directly correlated with take-over intention (the higher the score of PU and PEofU, the less likely they choose to take over), and this result verifies the positive effects of PU and PEofU on the use of automated driving technology found in relevant studies. There is a statistically significant path from PU to trust to AV take-over intention; recognition of the functions of AVs enhances drivers’ trust in the technology, thus weakens their take-over intention. The path from self-efficacy to trust to AV take-over intention, and that from likelihood of a crash to trust to AV take-over intention are statistically significant. The higher the score of self-efficacy or likelihood of a crash the stronger or weaker trust in automated driving. This suggests that drivers with higher self-efficacy scores are more confident in their driving skills, hence have a greater intention to be in control of the vehicle.

In summary, our study draws the following conclusions and suggestions. Among the demographic factors, age and educational level influence drivers’ take-over intention. As their age increases, drivers become more confident to take over the control of the vehicle when the perceived risk enhances. Drivers with higher education are more willing to trust automated driving technology and relinquish the control of AVs when it is safe to do so, possibly due to a better understanding of the technology. Therefore, this paper argues that it is important to provide essential training for correct AV take-over behavior. This is supported by the results from Payre’s simulator experiments, which have proved that automated driving training can improve the manual control recovery ability of nonprofessional drivers [42]. Such training should place more emphasis on a sound understanding of the functional range of AVs, so drivers can fully recognize when it is safe to relinquish the control and when they must take back the control. In addition, the possibility that a driver will relinquish control should be fully considered in AV design to improve the handling ability of AVs during emergencies (for example, if the driver fails to respond to a take-over request within a pre-defined period of time, the AV should activate the automatic emergency measures to slow down and then stop the vehicle at a safe spot. Moreover, the PE and NS results indicate that drivers’ psychological and physiological status can also impact on their take-over intention. Different from environmental and road-related take-over scenarios, it seems that, under PE and NS, drivers take back the control of the vehicle to enjoy driving or relinquish the control to relax. Their intention is not linked to their trust in technology. Taking such a human factor in the design will enable the technology to be more user friendly. It is also important that the automated driving system should check upon the driver’s alertness periodically by inviting them to participate in the task of driving. At which point, the driver may choose to drive or not). The automated driving system should aim to improve the driver’s situational awareness so they can deal with emergencies safely. Under the influence of the external environment (CC and RC), the results of the model show that in the face of possible risk scenarios, trust, PU and PEofU are important factors that affect drivers’ judgment with regard to take-over, whilst self-efficacy and likelihood of a crash are indirect influencing factors.

A few limitations remain with our study. First, due to the low penetration rate of AVs, all of the drivers in this study had not operated or ridden in an SAE level 3 vehicle. To level the understanding of the technology among the participants as much as possible, we showed them video segments about self-built AV driving prior to administering the questionnaire surveys. Second, this study adopted a self-report method to obtain personal information on number of accidents, health and driving style. Thus, the accuracy of such data depends on the participants’ willingness to provide true information. Third, a pure survey with questionnaires can of course only capture the subjective opinions of the respondents. In the future, we will validate our results with driving simulator experiments.

## Figures and Tables

**Table 1:** Statistical table of basic information of respondents.

Variable	*N*	Percentage
Gender		
Man	226	81.6%
Woman	51	18.4%
Degree of education		
Below bachelor’s degree	95	34.3%
Bachelor’s degree	165	59.6%
Postgraduate and above	17	6.1%
Have you used any driving assistance system?		
Yes	241	87%
No	36	13%
Level of Trust in automated driving		
Extremely low	10	3.6%
Quite low	46	16.6%
So-so	143	51.6%
Quite high	68	24.5%
Extremely high	10	3.6%
Age		
≤20	36	13.0%
21–30	154	55.6%
31–40	54	19.5%
≥41	31	11.9%
Driving years		
≤5	192	69.3%
6–10	51	18.4%
11–15	14	5.1%
>15	20	7.2%

**Table 2:** Factor loadings for the scale.

**Items**	**Factor1**	**M**	**S.D.**	**Items**	**Factor2**	**M**	**S.D.**
1	0.839	1.98	0.81	10	0.557	2.15	0.97
2	0.874	1.86	0.87	13	0.600	2.02	0.89
3	0.830	1.77	0.82	14	0.736	1.99	0.88
4	0.731	2.15	0.97	15	0.629	2.21	1.03
5	0.756	1.86	0.78	16	0.614	2.23	1.05
				18	0.770	2.29	1.03
				19	0.797	2.06	0.98
				20	0.809	2.34	1.02
				21	0.632	2.49	1.06
**Items**	**Factor3**	**M**	**S.D.**	**Items**	**Factor4**	**M**	**S.D.**
23	0.792	2.94	1.22	12	0.697	3.49	1.10
24	0.903	2.99	1.26	28	0.839	3.18	1.14
25	0.892	2.99	1.28	29	0.882	3.28	1.12
26	0.896	3.06	1.16				
27	0.870	3.06	1.23				

**Table 3:** The internal consistency.

	CC	RC	NS	PE	Total
*n*	5	9	5	3	22
Cronbach’s Alpha	0.886	0.901	0.954	0.830	0.924

**Table 4:** The correlation matrix between the factors of the scale and total score.

Factor	CC	RC	NS	PE	Total
CC	1.00				
RC	0.493 **	1.00			
NS	0.172 *	0.515 **	1.00		
PE	0.188 *	0.435 **	0.464 **	1.00	
Total	0.577 **	0.888 **	0.778 **	0.641 **	1.00

Notes: * *p* < 0.05; ** *p* < 0.01.

**Table 5:** Model adaptation indexes.

Index	CMIN/DF	TLI	CFI	RMSEA
Evaluation Standard	<3	>0.9	>0.9	<0.1
Measured Value	2.104	0.901	0.913	0.090

**Table 6:** TAM factor structure and factor loading.

**Items**	**M**	**S.D.**	**r**	**Factor Loading**
**Factor 1: PU**				
4. It saves time that I would have lost driving manually.	3.98	1.91	0.75 **	0.72
5. It prevents traffic violations.	4.45	1.73	0.78 **	0.70
6. The automated system in the car helps me with driving.	5.08	1.48	0.86 **	0.85
7. The automated system in the car enables me to drive well.	4.62	1.67	0.82 **	0.75
8. The automated system in the car system is useful to have.	5.42	1.32	0.76 **	0.74
**Factor 2: PEofU**				
9. The automated system in the car is clear and understandable.	4.76	1.37	0.83 **	0.80
10. The automated system in the car does not require a lot of mental effort.	3.80	1.73	0.81 **	0.77
11. The automated system in the car is easy to use.	4.57	1.40	0.87 **	0.86
12. The automated system in the car does what I want.	4.42	1.49	0.83 **	0.74

Notes: ** *p* < 0.01.

**Table 7:** ADSES factor structure and factor loading.

Item	M	S.D.	r	Factor Loading
Self-efficacy				
1. Driving in your local area	5.34	2.42	0.58 **	0.57
2. Driving in heavy traffic	5.97	2.45	0.83 **	0.83
3. Driving in unfamiliar areas	7.01	2.33	0.77 **	0.76
4. Driving at night	7.39	2.18	0.84 **	0.84
5. Driving with people in the car	6.97	2.24	0.85 **	0.85
6. Responding to road signs/traffic signals	6.71	2.34	0.81 **	0.82
7. Driving around a roundabout	6.59	2.43	0.85 **	0.85
8. Attempting to merge with traffic	6.11	2.35	0.87 **	0.87
9. Turning right across oncoming traffic	7.16	2.43	0.84 **	0.84
10. Planning travel to a new destination	7.59	2.16	0.80 **	0.80
11. Driving in high-speed areas	7.16	2.42	0.75 **	0.75
12. Parallel parking	7.59	2.16	0.70 **	0.69

Notes: ** *p* < 0.01.

**Table 8:** Correlation between RPS3 subscales and aggregate tables.

RPS	Worry and Insecurity α = 0.875	Likelihood of Crash α = 0.857	Concern α = 0.871	Total α = 0.862
Worry and insecurity (M = 3.34; SD = 1.00)	1.00			
Likelihood of crash (M = 2.63; SD = 0.77)	0.49 **	1.00		
Concern (M = 3.830; SD = 0.96)	0.40 **	0.15 *	1.00	
Total (M = 3.153; SD = 0.89)	0.88 **	0.77 **	0.58 **	1.00

Notes: * *p* < 0.05; ** *p* < 0.01.

**Table 9:** Relationship between trust, technology acceptability, demographic factors and automated driving take-over intention.

	CC	RC	NS	PE
Trust	−0.32 **	−0.25 **	−0.11	−0.21 **
PU	−0.39 *	−0.48 **	−0.57 **	−0.46 **
PEofU	−0.41 **	−0.46 **	−0.43 **	−0.34 **
Self-efficacy	0.06	−0.05	0.03	−0.10
Worry and insecurity	−0.03	−0.09	−0.12	−0.10
Likelihood of crash	−0.07	−0.08	−0.18 **	−0.53
Concern	−0.09	−0.12	−0.15 *	−0.14 *
Gender	−0.05	0.06	0.12	0.15 *
Age	0.11	0.06	0.24 **	0.11
Education	−0.10	−0.20 **	−0.22 **	−0.15 *
Driving experience	0.13 *	0.05	0.16 **	0.07

Notes: * *p* < 0.05; ** *p* < 0.01.

**Table 10:** Relationship between trust and technology acceptance, risk perception, self-efficacy.

	PU	PEOFU	Worry	Likelihood of Crash	Concern	Self-Efficacy
Trust	0.25 **	0.23 **	−0.51	−0.13 **	0.03	0.17 **

Notes: ** *p* < 0.01.

**Table 11:** Differences of take-over intention among age groups.

M (SD)	Age under-20 (*N* = 36)	Age 21–30 (*N* = 154)	Age 31–40 (*N* = 54)	Age over-41 (*N* = 33)	F
CC	3.9 (0.78)	4.03 (0.72)	4.24 (0.60)	4.18 (0.72)	1.98
RC	3.79 (0.81)	3.75 (0.77)	3.95 (0.64)	3.81 (0.87)	0.93
NS	2.46 (0.97)	2.93 (1.17)	3.34 (0.95)	3.28 (1.25)	5.36 **
PE	2.60 (0.84)	2.60 (0.94)	3.04 (1.00)	2.59 (1.08)	3.06 *

Notes: * *p* < 0.05; ** *p* < 0.01.

**Table 12:** Differences of take-over intention among education groups.

M (SD)	Below Bachelor’s Degree (*N* = 95)	Bachelor’s Degree (*N* = 165)	Master’s Degree or above (*N* = 17)	F
CC	4.15 (0.73)	4.07 (0.68)	3.75 (0.85)	2.23
RC	4.00 (0.72)	3.71 (0.77)	3.58 (0.67)	5.47 **
NS	3.43 (1.14)	2.75 (1.08)	2.89 (1.11)	11.56 ***
PE	2.90 (1.02)	2.56 (0.94)	2.69 (0.68)	3.90 *

Notes: * *p* < 0.05; ** *p* < 0.01; *** *p* < 0.001.

**Table 13:** Table of standardized path analysis data.

	Path	CC	RC
1	PEofU→Take-over	−0.256 ***	−0.248 ***
2	PU→Trust	0.262 ***	0.262 ***
3	PU→Take-over	−0.182 **	−0.305 **
4	self-efficacy→Trust	0.123 *	0.123 *
5	Likelihood of crash→Trust	−0.138 *	−0.138 *
6	Trust→Take-over	−0.217 ***	−0.113 ***

Notes: * *p* < 0.05; ** *p* < 0.01; *** *p* < 0.001. →: The arrow connects two variables, reflecting the causal relationship between the two.

## Data Availability

Data will be made available on reasonable request from the corresponding author.
